# Supplementary material for: Active conformation of the p97-p47 unfoldase complex
Source: Nat Commun. 2022 May 12;13:2640. doi: 10.1038/s41467-022-30318-3 (PMC9098461; doi:10.1038/s41467-022-30318-3)
Supplement: Supplementary file 1 — Supplementary Information [file 41467_2022_30318_MOESM1_ESM.pdf]

Supplementary Table 1

**Structure**	p97-p47 substrate-bound class	p97-p47 substrate-free class
EM Databank Accession ID	EMD-23835	EMD-26654
Protein Data Bank Accession ID	PDB 7MHS	
**Data collection**		
Microscope	Titan Krios	Titan Krios
Voltage (kV)	300	300
Detector	Gatan K3	Gatan K3
Data collection software	SerialEM	SerialEM
Magnification	81,000x	81,000x
Dose rate (e^-/Å^2/second)	17.1	17.1
Total number of frames	40	40
Total electron exposure (e^-/Å^2)	46	46
Defocus range (µm)	-1.0 to -2.2	-1.0 to -2.2
Pixel size (Å)	0.54 (super-resolution)	0.54 (super-resolution)
**Data processing**		
Number of micrographs	9,732	9,732
Initial particle images	1,519,419	1,519,419
Symmetry imposed		
3D classification	C1	C1
3D refinement	C1	C6
Final particle images	85,965	24,013
Map resolution (Å)		
FSC 0.143 (unmasked)	4.2	7.0
FSC 0.143 (masked, corrected)	3.6	5.7
**Model Refinement**		
Initial model used (PDB code)	5FTN	
Map sharpening B factor (Å^2)	-127	
Map correlation coefficient	0.78	
Model composition		
Non-hydrogen atoms	21,148	
Protein residues	2,674	
Ligands (ADP, BeF_x, Mg^{2+})	26	
R.m.s. deviations		
Bond lengths (Å)	0.004	
Bond angles (°)	0.934	
Validation		
MolProbity score	1.70 (100%)	
Clashscore	7.33 (100%)	
Poor rotamers (%)	0.00	
Ramachandran plot		
Favored (%)	95.65	
Allowed (%)	4.35	
Disallowed (%)	0	
C-beta deviations (0.25 Å)	0	
CaBLAM outliers (%)	3.3	
EMRinger Score	0.92	

Supplementary Table 1. Cryo-EM data collection, refinement, and validation statistics.

Supplementary Table 2

Ub linkage	Peptide	-10logP	Mass	Length	ppm	m/z	Z	RT	AScore
K6	MQIFVK(+114.04) TLTGK.T	16.26	1378.764	11	4.9	690.3928	2	48.37	7.21
K11	K.TLTGK(+114.04) TITLEVEPSDTIEN VK.A	71.4	2401.259	21	6.9	1201.645	2	66.49	11.1
K48	R.LIFAGK(+114.04) QLEDGR.T	59.79	1459.778	12	0.6	730.8969	2	38.9	1000
K48	R.LIFAGK(+114.04) QLED.G	19.34	1246.656	10	6.9	624.3395	2	56.59	1000
K48	L.IFAGK(+114.04) QLEDGR.T	37.49	1346.694	11	3.2	674.3565	2	38.92	1000
K48	I.FAGK(+114.04) QLEDGR.T	36.08	1233.61	10	1	617.8129	2	39.91	1000
K63	R.TLSDYN IQK(+114.04) ESTLHLVLR.L	68.39	2243.191	18	5.2	1122.609	2	65.47	48.87

Supplementary Table 2. IP-MS of observed ubiquitin peptides (Uniprot accession # P0CG47).

Supplementary Figure 1

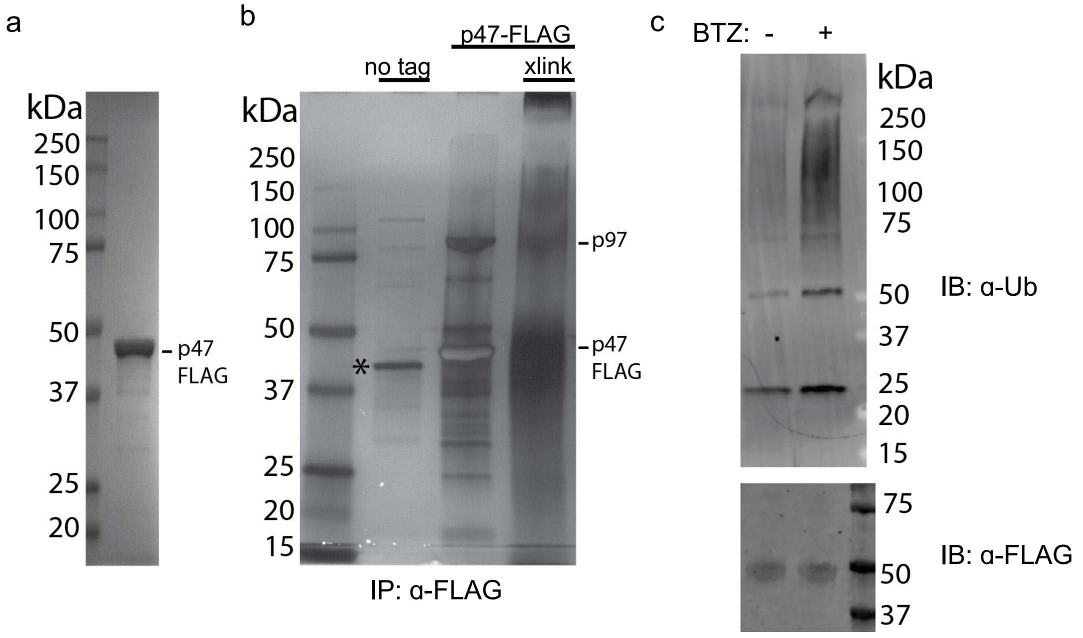

Supplementary Figure 1. Purification of p97 complexes.
Each experiment was repeated independently at least three times with similar results.
a, Coomassie-stained SDS-PAGE of purified His-p47-FLAG. (kDa molecular weights indicated)
b, Silver stained SDS-PAGE of anti-FLAG co-IPs. Lane assignments, left to right: ladder (kDa molecular weights indicated); eluate of α-FLAG co-IP from HEK293S cell lysates without addition of recombinant p47; eluate of α-FLAG co-IP with p47-FLAG added to lysates; p47-FLAG eluate following glutaraldehyde crosslinking used for cryo-EM grid preparation. Asterisk indicates contaminant actin band that is prominent in the control purification in the absence of p47, but not in the purification with added p47, presumably because high affinity p47-FLAG displaces actin that associates non-specifically with the α-FLAG resin (see also Supplementary Fig. 8).
c, Anti-ubiquitin and anti-FLAG immunoblots of p47-FLAG eluates from HEK293S cells with (+) or without (-) bortezomib (BTZ) treatment. Full scan blot images available in Source Data file.

Supplementary Figure 2

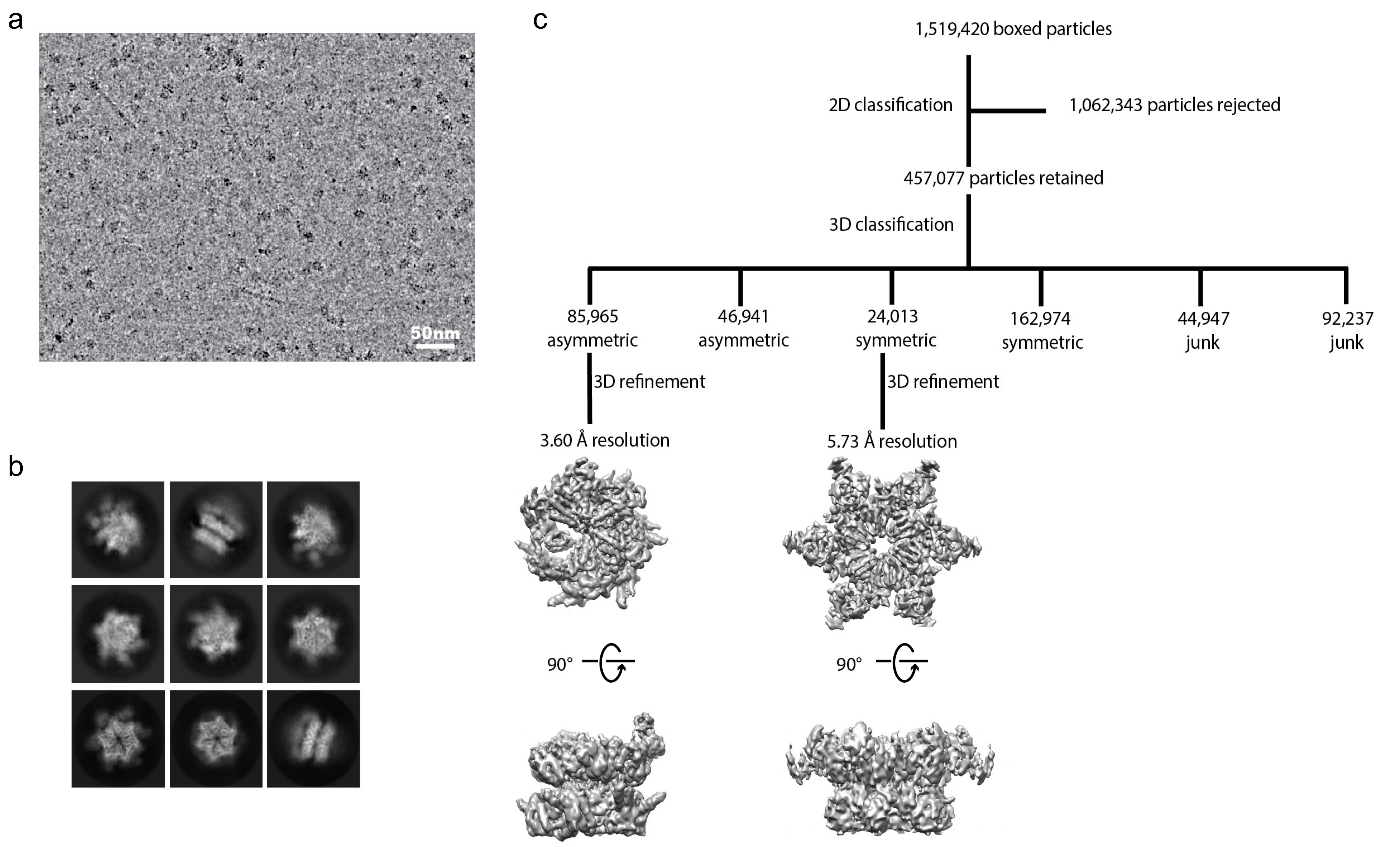

Supplementary Figure 2. Cryo-EM of p97-p47 complexes purified in the presence of ADP·BeFx.
a, Representative cryo-EM micrograph of p47 co-IP eluate. A total of 9,732 micrographs were recorded.
b, Subset of reference-free 2D class averages.
c, Data processing workflow. Full details in Methods.

Supplementary Figure 3

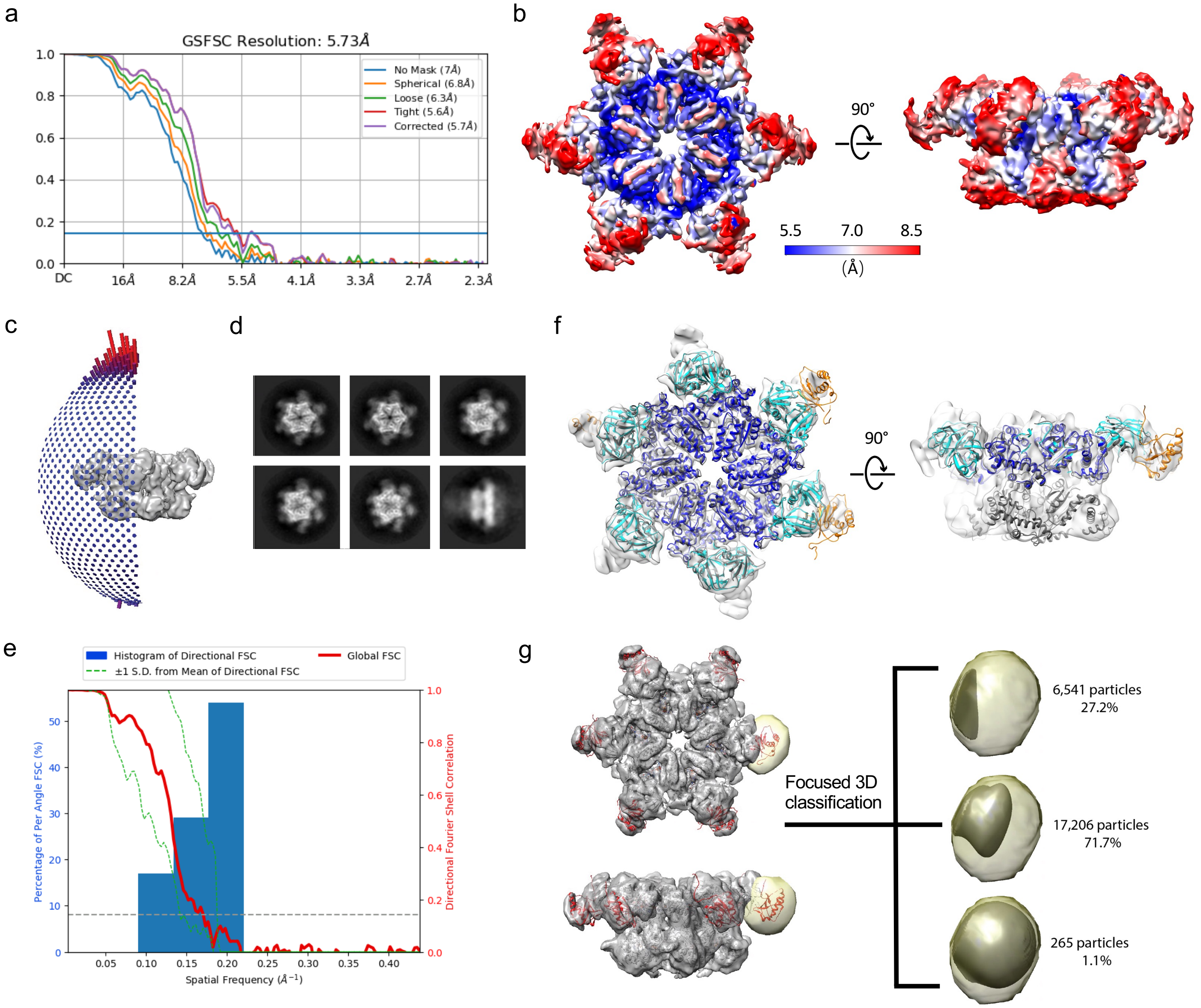

h

Class	Particles with UBX occupancy	Particles Without UBX occupancy	Percent Occupancy
1	18290	5723	76.2%
2	17207	6806	71.7%
3	21720	2293	90.5%
4	19283	4730	80.3%
5	14235	9778	59.3%
6	22220	1793	92.5%

Supplementary Figure 3. Reconstruction and classification of the substrate-free p97-p47 complex.
a, Gold standard FSC plots.
b, Local resolution heat map.
c, Particle orientation assignments.
d, 2D class averages of particles classified into substrate-free 3D class.
e, Histogram and directional FSC plot (3DFSC output).
f, Reconstruction fitted with ADP-bound p97 (dark grey) and p47(UBX)-p97(N-D1) (UBX, orange; N-domain cyan; D1 dark blue).
g, Representative example of UBX focused classification. A soft mask was applied over each N domain.
h, Focused classification distribution of particles with and without UBX density for each subunit.

Supplementary Figure 4

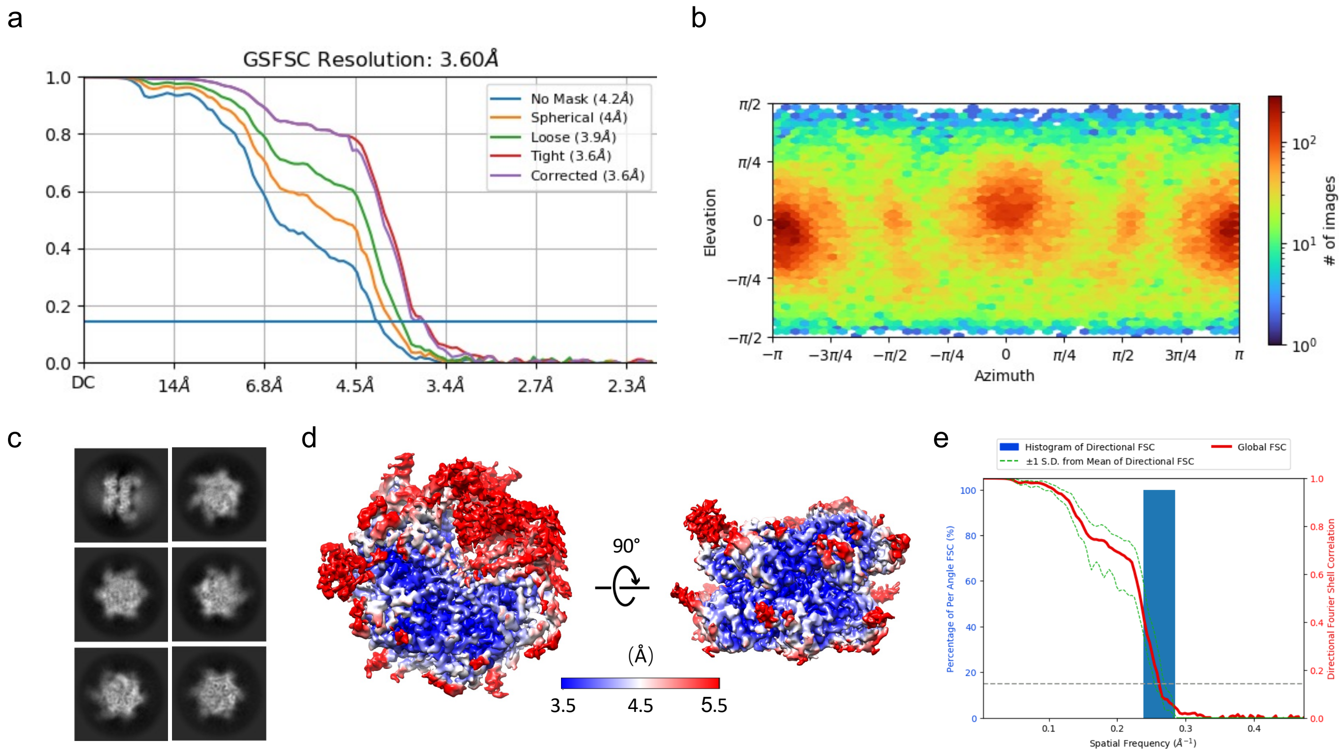

Supplementary Figure 4. Reconstruction of the substrate-bound p97-p47 complex.
a, Gold standard FSC plots.
b, Particle orientation assignments.
c, 2D class averages of particles classified into substrate-free 3D class.
d, Local resolution heat map.
e, Histogram and directional FSC plot (output from 3DFSC server).

Supplementary Figure 5

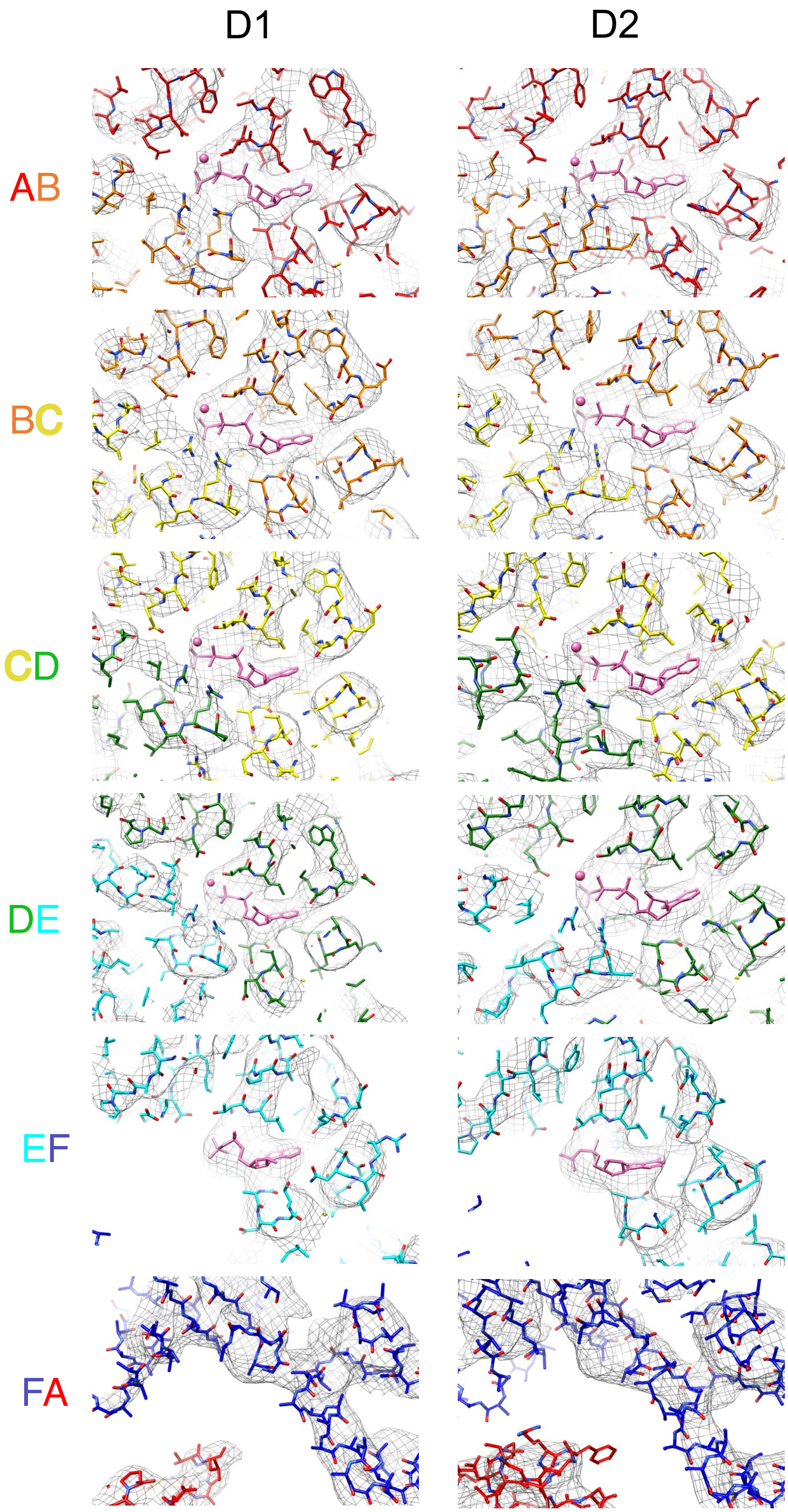

Supplementary Figure 5. Density and model around nucleotide binding pocket. The AB, BC, CD, and DE interfaces are modeled as ATP and Mg2+ (sphere). The EF interface is modeled as ADP. The FA interface is modeled without nucleotide (apo). All densities contoured to the same threshold level. Same coloring scheme used as in Fig. 2.

Supplementary Figure 6

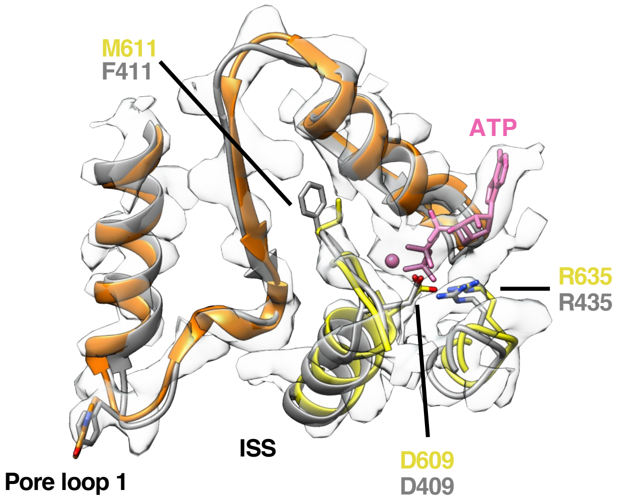

Supplementary Figure 6. Conservation of the ISS motif among substrate-bound AAA enzymes. Overlap of the ISS motif between YME1 (gray) and p97-D2 (subunit B, orange; subunit C, yellow) within p97 reconstruction density (transparent gray).

Supplementary Figure 7

a

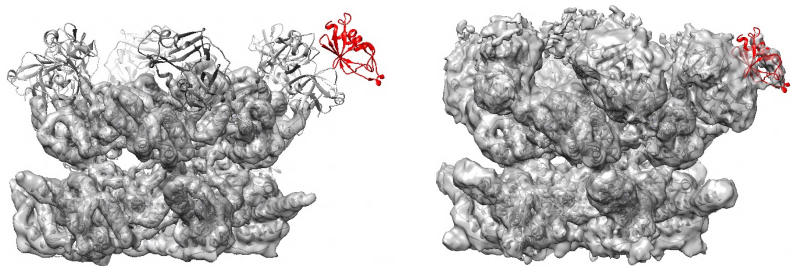

b

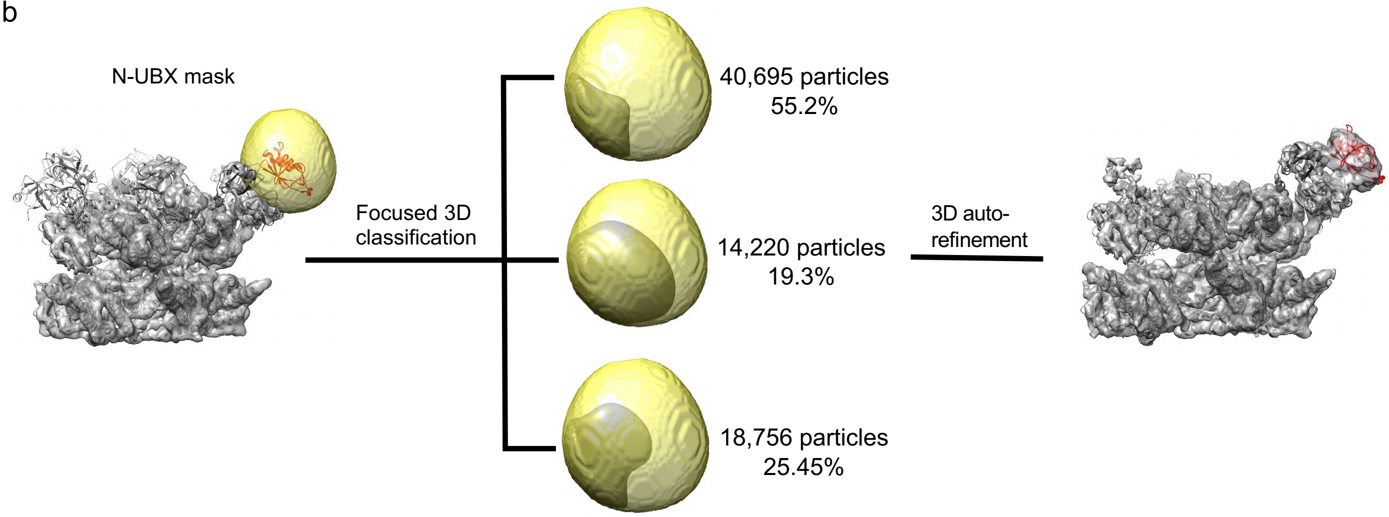

c

Subunit	Particles with UBX occupancy	Particles Without UBX occupancy	Percent Occupancy
A	10,169	63,532	13.8%
B	11,873	61,828	16.1%
C	14,220	59,481	19.3%
D	15,819	57,882	21.5%
E	16,395	57,306	22.2%
F	15,168	58,533	20.6%

d

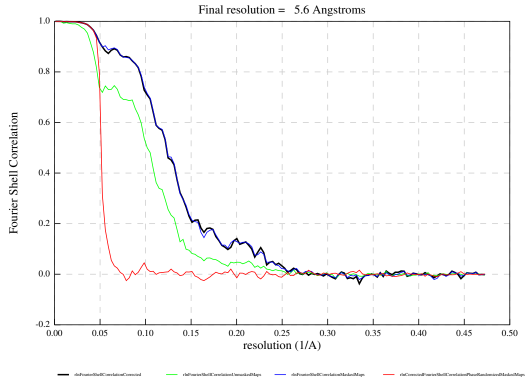

Supplementary Figure 7. Focused classification of p97(N)-p47(UBX) densities among substrate-bound p97 particles.

a, High- and low-threshold densities of the substrate-bound p97 reconstruction (left and right, respectively). The p47 UBX domain (red) is visible at low thresholds.

b, Focused classification scheme of the p97(N)-p47(UBX) densities. A soft-edge mask (yellow) was applied over the edge of the p97 N-domain and particles were classified without alignment. Class with most robust density was used as the basis for RELION auto-refinement. Subunit C shown here as representative example. Focused classification and auto-refinement were repeated for all subunits.

c, Distribution of particles with or without UBX density following focused 3D classification over each subunit.

d, Representative FSC plot for reconstruction of particles with UBX density for each subunit (subunit C shown here).

Supplementary Figure 8

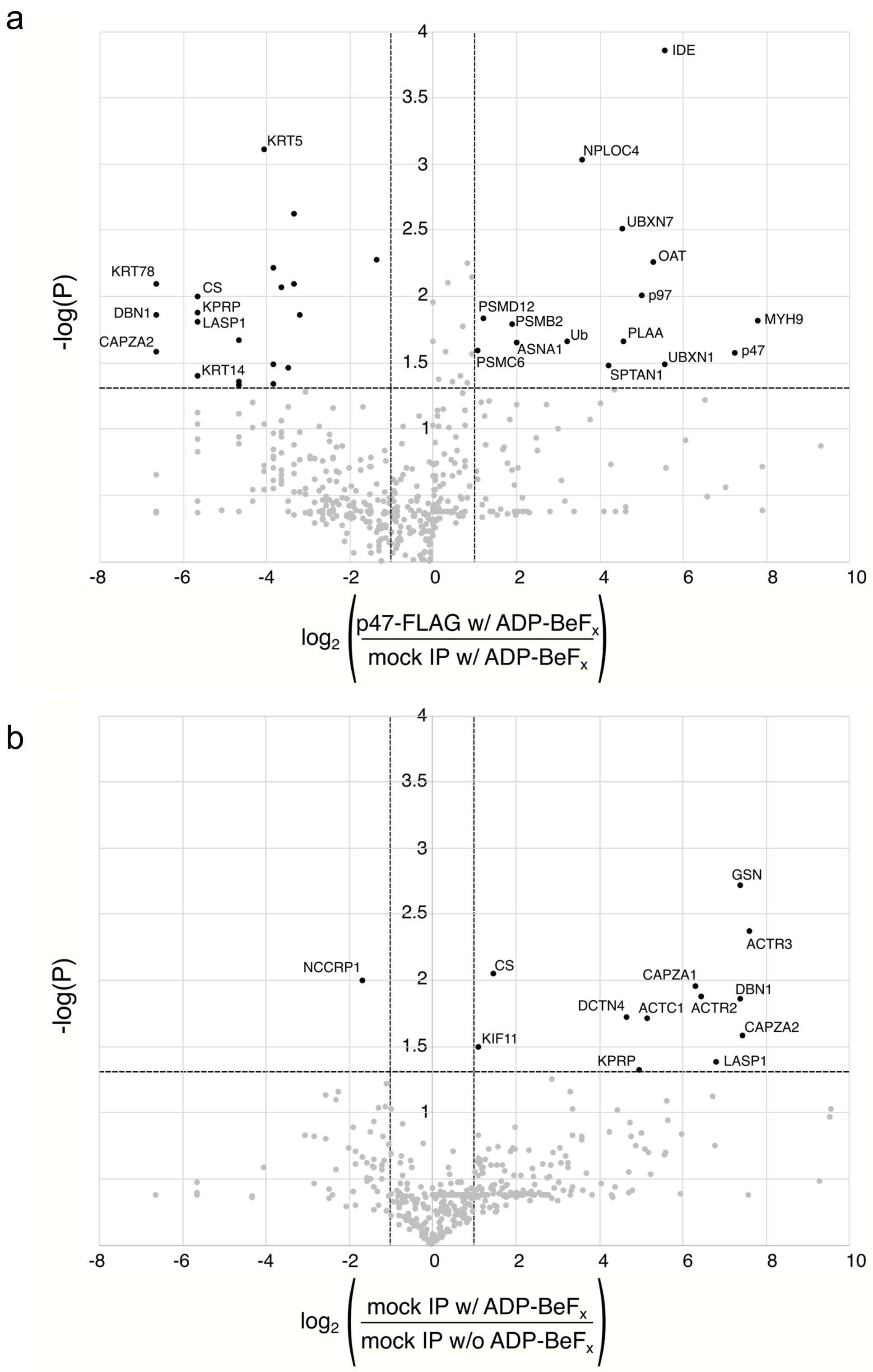

Supplementary Figure 8. LC-MS/MS identification and quantification of FLAG affinity purifications.
a, p47-FLAG coIPs in the presence of ADP·BeF$_x$ were compared against mock FLAG co-IPs of raw HEK293S lysates also in the presence of ADP·BeF$_x$. Volcano plot shows the log2 fold change of peptides detected in the p47-FLAG coIP relative to the mock IP control (x axis) and the -log(P) value of the detected hits (y axis).
b, Analysis of mock FLAG co-IPs performed with or without ADP·BeF$_x$ indicate a non-specific enrichment of actin-binding proteins in the presence of added nucleotide. Depletion of such proteins in panel a suggests these species were competed off the resin in the presence of FLAG co-IP.

Supplementary Figure 9

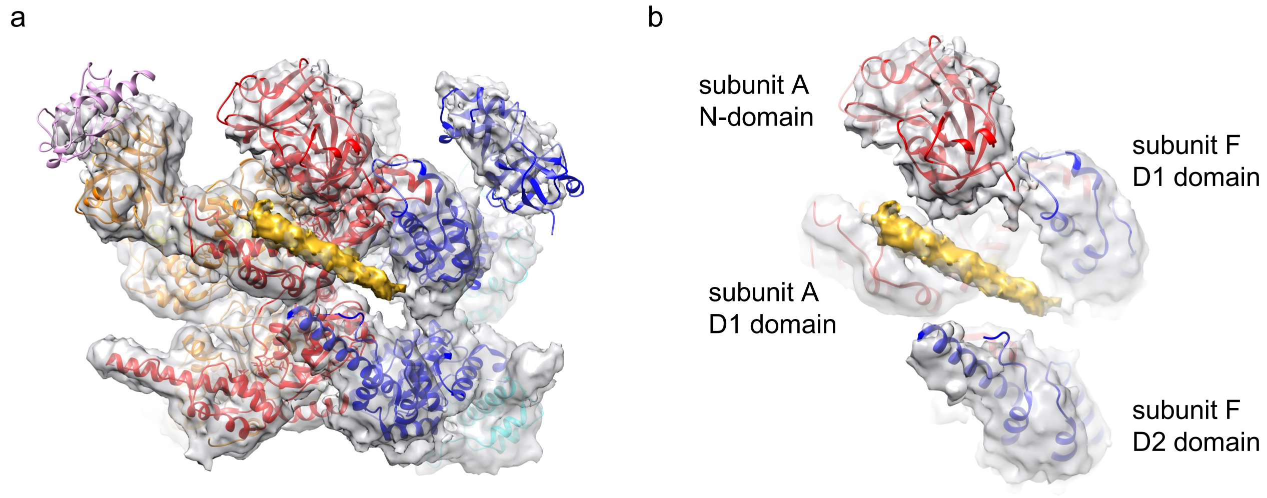

Supplementary Figure 9. Unidentified density at interface between subunits F and A.
a, Side view of the substrate-bound reconstruction facing the interface between subunit F (blue) and subunit A (red). Unidentified density segmented in orange.
b, Closeup view of the FA interface reveals an unmodeled elongated rod density (orange) spanning between the D1-D2 linker of subunit F and the N-D1 gap of subunit A.
