# Supplementary material for: Active conformation of the p97-p47 unfoldase complex
Source: Nat Commun. 2022 May 12;13:2640. doi: 10.1038/s41467-022-30318-3 (PMC9098461; doi:10.1038/s41467-022-30318-3)
Supplement: Supplementary file 3 — Reporting Summary [file 41467_2022_30318_MOESM3_ESM.pdf]

nature portfolio

Corresponding author(s): Peter S. Shen and Christopher P. Hill

Last updated by author(s): Apr 15, 2022

Reporting Summary

Nature Portfolio wishes to improve the reproducibility of the work that we publish. This form provides structure for consistency and transparency in reporting. For further information on Nature Portfolio policies, see our Editorial Policies and the Editorial Policy Checklist.

Statistics

For all statistical analyses, confirm that the following items are present in the figure legend, table legend, main text, or Methods section.

n/a	Confirmed	
☐	☒	The exact sample size (*n*) for each experimental group/condition, given as a discrete number and unit of measurement
☐	☒	A statement on whether measurements were taken from distinct samples or whether the same sample was measured repeatedly
☐	☒	The statistical test(s) used AND whether they are one- or two-sided<br>*Only common tests should be described solely by name; describe more complex techniques in the Methods section.*
☒	☐	A description of all covariates tested
☒	☐	A description of any assumptions or corrections, such as tests of normality and adjustment for multiple comparisons
☐	☒	A full description of the statistical parameters including central tendency (e.g. means) or other basic estimates (e.g. regression coefficient) AND variation (e.g. standard deviation) or associated estimates of uncertainty (e.g. confidence intervals)
☒	☐	For null hypothesis testing, the test statistic (e.g. F, t, r) with confidence intervals, effect sizes, degrees of freedom and P value noted<br>*Give P values as exact values whenever suitable.*
☒	☐	For Bayesian analysis, information on the choice of priors and Markov chain Monte Carlo settings
☒	☐	For hierarchical and complex designs, identification of the appropriate level for tests and full reporting of outcomes
☒	☐	Estimates of effect sizes (e.g. Cohen's *d*, Pearson's *r*), indicating how they were calculated

Our web collection on statistics for biologists contains articles on many of the points above.

Software and code

Policy information about availability of computer code

Data collection	Cryo-EM data were collected on ThermoFisher Titan Krios/Gatan K3 using SerialEM (v3.8).<br>Mass spectrometry data were collected on ThermoFisher Orbitrap Fusion Lumos.
Data analysis	Cryo-EM data were processed using cryoSPARC (v3.0) and RELION (v3.1). Model building performed in Coot (v0.8.7). Model refined using Phenix (v1.19). Model visualization and analysis using UCSF Chimera (ver. 1.15)<br>Mass spectrometry data were processed using PEAKS Studio software (version X pro).

For manuscripts utilizing custom algorithms or software that are central to the research but not yet described in published literature, software must be made available to editors and reviewers. We strongly encourage code deposition in a community repository (e.g. GitHub). See the Nature Portfolio guidelines for submitting code & software for further information.

Data

Policy information about availability of data

All manuscripts must include a data availability statement. This statement should provide the following information, where applicable:

- Accession codes, unique identifiers, or web links for publicly available datasets
- A description of any restrictions on data availability
- For clinical datasets or third party data, please ensure that the statement adheres to our policy

All maps have been deposited to the EM Databank and are accessible via accession numbers EMD-23835 (substrate-bound p97-p47) and EMD-26654 (substrate-free p97-p47). The coordinate models for substrate-bound p97-p47 are accessible on the Protein Data Bank via PDB ID 7MHS. Mass spectrometry data files have been deposited to the Chorus repository server (no. 1764).

Field-specific reporting

Please select the one below that is the best fit for your research. If you are not sure, read the appropriate sections before making your selection.

☒ Life sciences ☐ Behavioural & social sciences ☐ Ecological, evolutionary & environmental sciences

For a reference copy of the document with all sections, see nature.com/documents/nr-reporting-summary-flat.pdf

Life sciences study design

All studies must disclose on these points even when the disclosure is negative.

Sample size	A total of 1,519,419 cryo-EM particles were selected across 9,732 micrographs.<br>Mass spectrometry samples were processed in triplicate (n=3). The -logP values for each protein were calculated from Pvalues obtained using a two-way heteroscedastic t-test to compare the replicate measurements in each sample. No values were imputed for zeros, no multiple testing corrections were applied as the analysis relied on multiple criteria beyond p-value to establish significance.
Data exclusions	Cryo-EM particles sorted into unresolved (junk) 2D and 3D classes were excluded from final refinements. No other data were excluded.
Replication	Image processing of cryo-EM datasets has inherent replication. Mass spectrometry analyses were performed with three biological replicates for each sample. All attempts at replication were successful.
Randomization	Cryo-EM datasets are split into two random halves during 3D reconstruction. Mass spectrometry samples were processed in a block randomized fashion.
Blinding	Blinding is not applicable to cryo-EM experiments. Proteomics data were acquired in a blinded fashion.

Reporting for specific materials, systems and methods

We require information from authors about some types of materials, experimental systems and methods used in many studies. Here, indicate whether each material, system or method listed is relevant to your study. If you are not sure if a list item applies to your research, read the appropriate section before selecting a response.

Materials & experimental systems

n/a	Involved in the study
☐	☒ Antibodies
☐	☒ Eukaryotic cell lines
☒	☐ Palaeontology and archaeology
☒	☐ Animals and other organisms
☒	☐ Human research participants
☒	☐ Clinical data
☒	☐ Dual use research of concern

Methods

n/a	Involved in the study
☒	☐ ChIP-seq
☒	☐ Flow cytometry
☒	☐ MRI-based neuroimaging

Antibodies

Antibodies used	anti-FLAG clone M2 affinity resin (Sigma, Product No. A2220)<br>anti-ubiquitin clone E412J (rabbit monoclonal IgG, Cell Signaling Technology, Product No. 43124S)<br>anti-FLAG clone M2 (mouse monoclonal IgG, Sigma, Product No. F1804)<br>Goat anti-mouse IgG secondary antibody (LI-COR, Product No. 926-32210)<br>Goat anti-rabbit IgG secondary antibody (LI-COR, Product No. 926-68071)
Validation	All antibodies were tested by immunoblot with relevant positive controls.<br>Validation statements are available through the manufacturers' websites:<br>anti-FLAG: https://www.sigmaaldrich.com/deepweb/assets/sigmaaldrich/product/documents/144/194/vol6_iss2_antiflag_m2.pdf<br>anti-ubiquitin: https://www.cellsignal.com/products/primary-antibodies/ubiquitin-e4i2j-rabbit-mab/43124<br>Goat anti-mouse IgG secondary antibody: https://www.licor.com/bio/reagents/irdye-800cw-goat-anti-mouse-igg-secondary-antibody<br>Goat anti-rabbit IgG secondary antibody: https://www.licor.com/bio/reagents/irdye-680rd-goat-anti-rabbit-igg-secondary-antibody

Eukaryotic cell lines

Policy information about cell lines

Cell line source(s)	HEK293GnTI- (ATCC CRL-3022)

Authentication	Mass spectrometry of IP eluates confirmed peptides originated from human proteome
Mycoplasma contamination	Cells tested negative for mycoplasma contamination.
Commonly misidentified lines (See ICLAC register)	No commonly misidentified cell line was used in this study.
