# Supplementary figures and images for: Active conformation of the p97-p47 unfoldase complex
Source: Nat Commun. 2022 May 12;13:2640. doi: 10.1038/s41467-022-30318-3 (PMC9098461; doi:10.1038/s41467-022-30318-3)

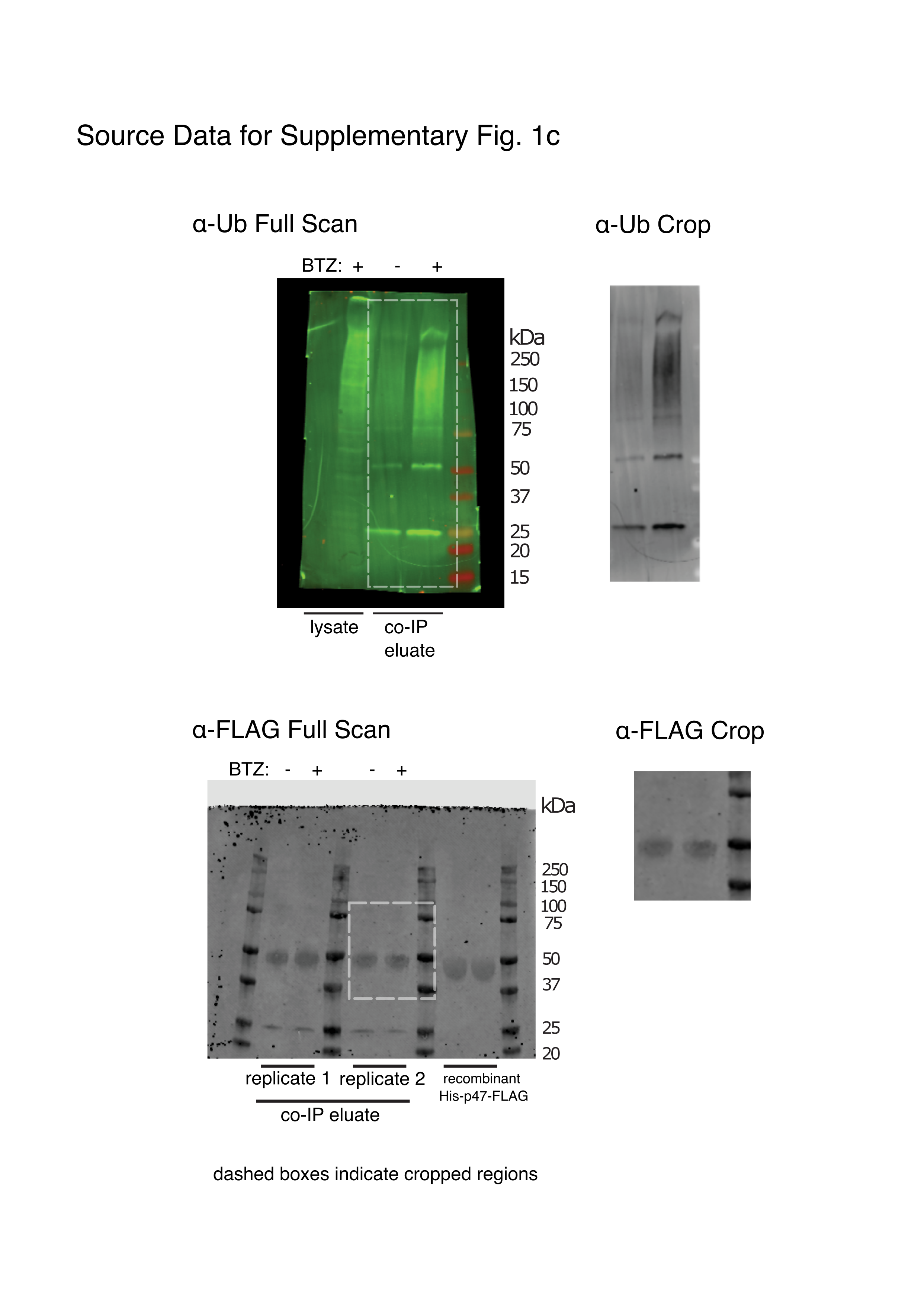

Supplement: Supplementary file 4 — Source Data [file 41467_2022_30318_MOESM4_ESM.png]
